# Human resource for health reform in peri-urban areas: a cross-sectional study of the impact of policy interventions on healthcare workers in Epworth, Zimbabwe

**DOI:** 10.1186/s12960-017-0260-x

**Published:** 2017-12-16

**Authors:** Bernard Hope Taderera, Stephen James Heinrich Hendricks, Yogan Pillay

**Affiliations:** 1 School of Health Systems and Public Health, University of Pretoria, 31 Bophelo Road, Gezina, Pretoria, South Africa; 2 Department of Political and Administrative Studies, University of Zimbabwe, P.O. Box MP 167, Mount Pleasant, Harare, Zimbabwe; 3 National Department of Health of the Republic of South Africa, Civitas Building, 222 Thabo Sehume St, Pretoria, South Africa

**Keywords:** Human resources, Health reform, Peri-urban, Policy, Epworth, Zimbabwe

## Abstract

**Background:**

The need to understand how healthcare worker reform policy interventions impact health personnel in peri-urban areas is important as it also contributes towards setting of priorities in pursuing the universal health coverage goal of health sector reform. This study explored the impact of post 2008 human resource for health reform policy interventions on healthcare workers in Epworth, a peri-urban community in Harare, Zimbabwe, and the implications towards health sector reform policy in peri-urban areas.

**Methods:**

The study design was exploratory and cross-sectional and involved the use of qualitative and quantitative methods in data collection, presentation, and analysis. A qualitative study in which data were collected through a documentary search, five key informant interviews, seven in-depth interviews, and five focus group discussions was carried out first. This was followed by a quantitative study in which data were collected through a documentary search and 87 semi-structured sample interviews with healthcare workers. Qualitative data were analyzed thematically whilst descriptive statistics were used to examine quantitative data. All data were integrated during analysis to ensure comprehensive, reliable, and valid analysis of the dataset.

**Results:**

Three main factors were identified to help interpret findings. The first main factor consisted policy result areas that impacted most successfully on healthcare workers. These included the deployment of community health workers with the highest correlation of 0.83. Policy result areas in the second main factor included financial incentives with a correlation of 0.79, training and development (0.77), deployment (0.77), and non-financial incentives (0.75). The third factor consisted policy result areas that had the lowest satisfaction amongst healthcare workers in Epworth. These included safety (0.72), equipment and tools of trade (0.72), health welfare (0.65), and salaries (0.55).

**Conclusions:**

The deployment of community health volunteers impacted healthcare workers most successfully. This was followed by salary top-up allowances, training, deployment, and non-financial incentives. However, health personnel were least satisfied with their salaries. This had negative implications towards health sector reform interventions in Epworth peri-urban community between 2009 and 2014.

## Background

Exploring the impact of human resource for health reform policy interventions on healthcare workers in peri-urban areas helps lay a foundation upon which health sector reform may be formulated and implemented towards addressing the global health workforce crisis and attainment of universal health coverage [1, 2]. Progress made through resolution WHA67.24 on Follow-up of the Recife Political Declaration on Human Resources for Health: renewed commitments towards universal health coverage adopted in May 2014 and the World Health Report of December 2016 on Working for Health and Growth: Investing in the health workforce has contributed towards the agenda articulated by the 2030 Global Health Workforce Strategy [3–5]. From this, whilst equitable distribution, availability, accessibility, competency, and motivation are priorities for health systems, this has also presented an opportunity to advance further towards more responsive human resource for health reform interventions in peri-urban areas [5]. One of the channels to advance towards responsive health sector reform is through the exploring how current human resources for health reform policy interventions impact healthcare workers in peri-urban areas [6]. Exploring the impact of human resource for health reform policy interventions in peri-urban areas also contributes towards the 2030 Sustainable Development Agenda, particularly goals 11, aimed at making cities and human settlements inclusive, safe, resilient, and sustainable, and 3, towards ensuring healthy lives and promoting well-being for all at all ages [7].

**Fig. 1:**
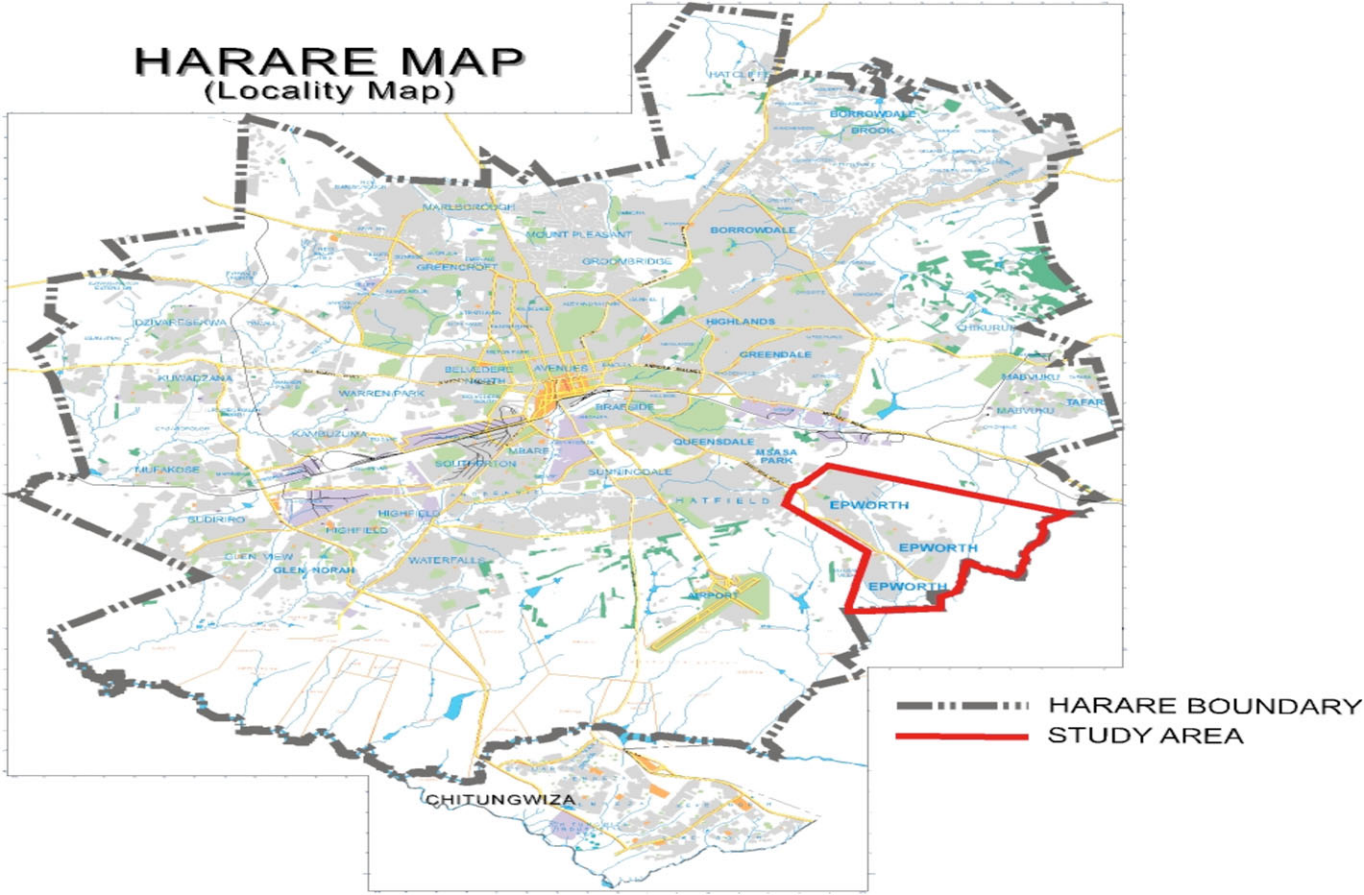
Location of Epworth in Harare. Reproduced with permission from Taderera et al. [8]

Peri-urban areas are a fringe located between the city and countryside that develop as a result of immigration from urban and rural areas thereby resulting in chaotic urbanization leading to a sprawl [8]. Epworth is a peri-urban area that developed as a result of rural-to-urban and urban-to-rural migration and is located on the south-east boundary of Harare as illustrated in Fig. 1.

**Fig. 2:**
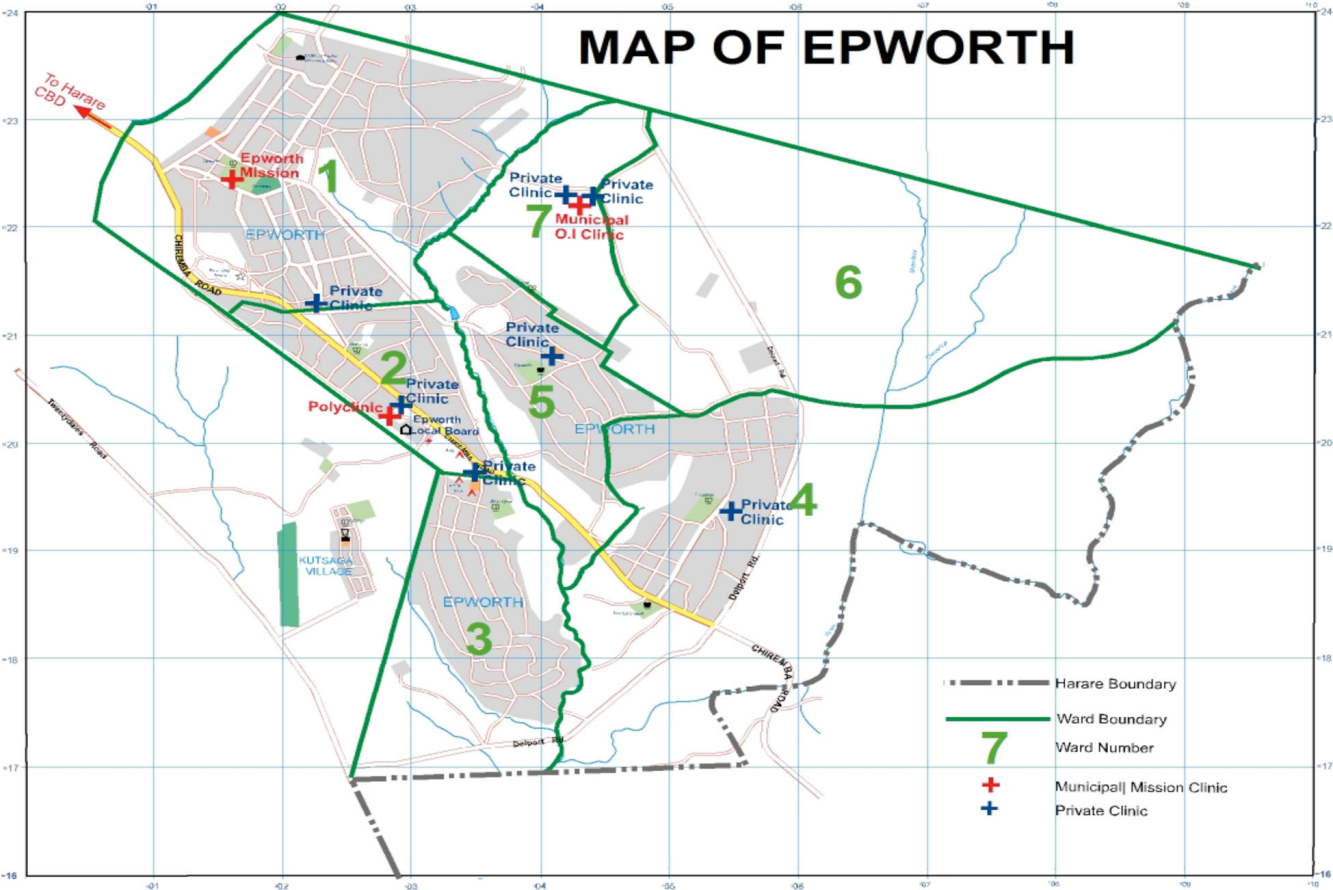
Map of Epworth. Reproduced with permission from Taderera et al. [8]

Initially, this area was established by Reverend Shimmin in 1890 as a Methodist Mission Station on a farm. Over the years, however, this area experienced an uncontrolled influx of people because of socio-political factors of the 1970s, the effects of austerity measures in the 1990s, and the prevailing socio-economic challenges of the new millennium [9]. Efforts by the Government of Zimbabwe to regularize this area have resulted in semi-formal settlement and the establishment of a Local Board, a form of municipal authority occupying the lowest position in the country’s local governance structure [9, 10]. From this regularization, Epworth has seven electoral wards within which there are seven small private clinics and three public health facilities, consisting of a Methodist Mission Clinic and two municipal clinics as illustrated in Fig. 2.

However, the continued influx of people into this area resulted in ever-increasing population and unplanned habitation on land that lacks basic amenities such as sewer and running water. This is compounded by the stiff contestation for scarce local resources which has resulted in impoverishment and contributed towards a very narrow revenue base and limited capacity by the Local Board. As a result, the area has a higher disease burden and is a potential disaster in terms of disease outbreaks [9].

For instance, in this context, the healthcare worker challenges that worsened between 2003 and 2008 because of the socio-economic challenges prevailing in Zimbabwe seriously affected peri-urban and rural communities [10]. Whilst the Ministry of Health intervened in 2009, our aim in this study was to explore the impact of the human resource for health policy interventions on healthcare workers. From this, we sought to drawn lessons through identifying policy priorities for future intervention towards health sector reform policy in peri-urban areas.

## Methods

### Research design

A cross-sectional survey design was used as it enabled the collection of data across Epworth community using qualitative and quantitative methods in data collection, presentation, and analysis. Multiple data sources were used to generate a valid, reliable, and comprehensive dataset [11, 12].

### Qualitative study

Firstly, we carried out a documentary search to explore the human resource for health reform policy interventions implemented to address the healthcare worker challenges of pre-2008 in Zimbabwe. Findings from the documentary search were used to develop a key informant interview guide. This interview guide was piloted through two key informant interviews with participants (key informants) drawn from the Ministry of Health. The interview guide was then refined and used to carry out five more key informant interviews with purposively selected participants (key informants) drawn from the Ministry of Health (MoH), Health Services Board (HSB), Zimbabwe Association of Church Hospitals (ZACH), the Provincial Medical Office (PMO) in Marondera, and Epworth Local Board (ELB). A digital audio recorder, notebooks, and pens were also other equipment used. Data collected at this stage were used to develop an interview guide used to carry out seven in-depth interviews with purposively selected health personnel managers at local health clinics. These in-depth interviews were carried out to explore the impact of human resource for health reform interventions at the health clinic level. Data were also collected from community members who participated in five focus group discussions that consisted of 10 people each. The focus group discussions were carried out to explore outcomes of the human resource for health reform policy interventions on locals. The materials used in this qualitative study included an interview guide, digital audio recorders, pens, and notebooks. Data were collected from each category of participants until saturation was reached. The collection of data through key-informant interviews, in-depth interviews, and focus group discussions enabled us to triangulate our data sources so as to generate a valid, reliable, and comprehensive dataset [12]. Each interview in this qualitative study lasted between 30 and 45 min.

### Quantitative study

In order to be able to assess the impact of human resource for health reform interventions, our first task was to carry out a documentary search of staff registers to determine the total number of, and the sampling frame for, healthcare workers at clinics in Epworth. From the staff registers at each clinic in Epworth, it was established that there were 101 healthcare workers in Epworth as outlined in Table 1.

**Table 1:** Staff establishment at health facilities in Epworth

Facility type	Human resource for health managers	Nursing staff	Other cadres	Total for all cadres (excluding medical doctors and sisters in charge)
Mission clinic	1 sister in charge	2 primary counselors, 6 registered general nurses, and 2 primary care nurses.	1 environmental health officer/technician and 4 nurse aides	15
Municipal “polyclinic” clinic	1 sister in charge	11 registered general nurses, 6 midwives, 1 state certified nurse, 3 primary care nurses, and 2 primary counselors.	1 pharmacy technician, 3 laboratory scientists, 3 ambulance drivers, and 1 environmental health officer, 11 nurse aides	42
Municipal “OI” clinic	1 sister in charge	13 registered general nurses.	1 dispensary assistant 1 environmental technician, 5 nurse aides, and 1 pharmacy technician	21
Private clinic	1 general practitioner.	1 registered general nurse and 1 primary care nurse	2 nurse aides	4
Private clinic	1 general practitioner.	1 registered general nurse.	3 nurse aides, 1 lab pathologist 1 radiologist, and 1 dental surgeon	7
Private clinic	1 general practitioner.	1 registered general nurse and 1 midwife	2 nurse aides	4
Private clinic	1 general practitioner.	2 registered general nurses	1 nurse aide	3
Private clinic	1 general medical practitioner	1 primary care nurse	0	1
Private clinic	1 general practitioner.	2 registered general nurses	0	2
Private clinic	1 general practitioner		2 nurse aides	2
Total		56	45	101

Data generated from staff registers at local clinics

The sampling frame of these healthcare workers consisted of two main categories of health cadres namely medical and non-medical personnel. The medical personnel were nurses (registered general nurses, state certified midwives, and primary care nurses). The non-medical personnel included nurse aides, primary counselors, environmental health officers, pharmacy technicians, laboratory technicians, and ambulance drivers. Using the total population of 101 healthcare workers, we then used Taro Yamane’s Formula below for calculating the sample size [13]:

$$ \frac{n=N}{1+N{(e)}^2} $$

where

*n =* sample size

*N* = Population (i.e., 101)

*e* = the level of precision (i.e., 0.04)

Note: the level of precision used (0.04) is based on the confidence level of 96% that we set [13, 14].

In this regard, the sample size was then calculated as follows:

$$ \frac{n=101}{1+101{(0.04)}^2} $$

From this, *n* equals to 86.949. Rounding this up gave us a sample size of 87. From this, the proportionate number of sample interviews carried out with cadres at each clinic was then determined by proportionate distribution of health cadres at each clinic as outlined in Table 2.

**Table 2:** Proportionate distribution of health workers by facility

Facility type	Nursing staff	Other cadres	Total for all cadres	Proportion of the total sample size of 87 (%)	Total number of interviews
Mission clinic	2 primary counselors, 6 registered general nurses, and 2 primary care nurses	1 environmental health officer/technician and 4 nurse aides	15	15	13
Municipal “polyclinic” clinic	11 registered general nurses, 6 midwives, 1 state certified nurse, 3 primary care nurses, 2 primary counselors.	1 pharmacy technician, 3 laboratory scientists, 3 ambulance drivers, 1 environmental health officer, and 2 nurse aides	42	42	37
Municipal “OI” clinic	13 registered general nurses	1 dispensary assistant, 1 environmental technician, 1 pharmacy technician and 5 nurse aides	21	21	18
Private clinic	1 registered general nurse and 1 primary care nurse	2 nurse aides	4	3	3
Private clinic	1 registered general nurse	3 nurse aides 1 lab technician, 1 radiologist, and 1 dental surgeon	7	7	6
Private clinic	1 registered general nurse and 1 midwife	2 nurse aides	4	4	4
Private clinic	2 registered general nurses	1 nurse aide	3	3	3
Private clinic	1 primary care nurse	0	1	1	1
Private clinic	2 registered general nurses	0	2	2	1
Private clinic		2 nurse aides	2	2	1
Total	56	45	101		87

Data generated from staff registers at local clinics

This proportionate distribution of healthcare workers helped us determine the number of interviews carried out at each clinic as outlined in Table 3.

**Table 3:** Proportionate distribution of interviews by health worker category at each clinic

Facility type	Medical nursing staff and number of interviews	Other cadres and number of interviews
Mission clinic	6 registered general nurses (5 interviews) and 2 primary care nurses (2 interviews)	1 environmental health officer/technician (1 interview), 2 primary counselors (2 interviews), and 4 nurse aides (3 interviews);
Municipal “polyclinic” clinic	11 registered general nurses (10 interviews), 6 midwives (5 interviews), 1 state certified nurse (1 interview), and 3 primary care nurses (2 interviews)	1 pharmacy technician (1 interview), 3 laboratory scientists (3 interviews), 3 ambulance drivers (3 interviews), 1 environmental health officer (1 interview), 11 nurse aides (10 interviews), and 2 primary counselors (2 interviews)
Municipal “OI” clinic	13 registered general nurses (12 interviews)	1 dispensary assistant (1 interview), 1 environmental technician (1 interview), 1 pharmacy technician (1 interview), and 5 nurse aides (3 interviews)
Private clinic	1 registered general nurse (1 interview) and 1 primary care nurse (1 interview)	2 nurse aides (1 interview)
Private clinic	1 registered general nurse (1 interview) and 1 dental surgeon (1 interview)	3 nurse aides (2 interviews), 1 lab technician (1 interview), and radiologist (0 interview)
Private clinic	1 registered general nurse (1 interview); 1 midwife (1 interview).	2 nurse aides (2 interviews)
Private clinic	2 registered general nurses (2 interviews)	1 nurse aide (1 interview)
Private clinic	1 primary care nurse (1 interview)	0
Private clinic	2 registered general nurses (1 interview)	0
Private clinic		2 nurse aides (1 interview)
Total	47	40

Data generated from staff registers at local clinics

From this, we carried out semi-structured interviews with a sample of 87 healthcare workers at clinics across Epworth. A semi-structured questionnaire was used for this purpose, and each interview lasted between 30 and 45 min. Trustworthiness (credibility, transferability, dependability, and reliability) of data was ensured through making prior arrangements with respondents to interview them at their convenience and the use of the same data collection tool (semi-structured questionnaire) on all respondents in this category (for example, the category of healthcare workers at each clinic). In addition, the comparison of qualitative and quantitative data from each semi-structured interview in this category of healthcare workers at each clinic enabled us to cross-verify data for trustworthiness. The dataset from each category of participants was compared with that from other categories, for example, other categories that include healthcare worker managers at each local clinic and community members [12].

### Analysis of findings

Qualitative data were first transcribed to create narratives. The narratives were then coded manually to help identify the main categories of common narrations into which qualitative data were then put. We then determined the theme for each category before subjecting the data in that theme to interpretive thematic analysis [11]. The themes were then integrated with quantitative data analyzed using descriptive statistics for cross-verification (trustworthiness), comprehensive, reliable, and valid analysis. Descriptive statistics were used to analyze quantitative data using Statistical Package for Social Studies (SPSS). In this, we used an ordinal scale measurement level to assess the impact of human resources for health reform interventions on healthcare workers focusing on policy result areas. Using a Scree Plot, we were then able to identify the number of key factors into which variables were clustered. We then subjected these factors to correlation analysis to determine the levels of impact of each on healthcare workers. We then integrated quantitative data with qualitative narratives for comprehensiveness, reliability, and validity in analysis [11, 12].

### Authorization and research ethics clearance

This paper was generated from the dataset of a larger PhD in Public Health study carried out in Epworth, Zimbabwe. This study received institutional approval from the Academic Advisory Committee at the University of Pretoria. Institutional authorization was sought and granted by the Ministry of Health and Child Care of Zimbabwe, Health Services Board, Mashonaland East Provincial Medical Directorate, Seke District Medical Office, Epworth Local Board, and Zimbabwe Republic Police. Ethics clearance was sought and granted by the Research Ethics Committee of the Faculty of Health Sciences, University of Pretoria (Reference number 413/2014), and the Medical Research Council of Zimbabwe (Approval Number MRCZ/A/1941). Written informed consent to participate and for publication was sought and granted by all participants [9].

## Results

### Healthcare worker reform policy interventions

The Ministry of Health adopted the Human Resources for Health Policy that was implemented through the Human Resources for Health Strategic Plan between 2009 and 2014 to address the healthcare worker challenges experienced before 2008. Inquiry revealed policy result areas that included financial incentives, non-financial incentives, support for basic and post-basic training, health and safety welfare, deployment of adequate staff and workload, deployment of community health volunteers, provision of equipment and tools of trade, and salaries. We used an ordinal scale measurement level to assess the impact of these human resources for health reform policy interventions on healthcare workers in each of these policy result areas, and this yielded outcomes that are outlined in Table 4.

**Table 4:** Assessment levels of healthcare worker satisfaction

	All	Medical staff	Non-medical
Satisfaction assessment measures	*N* = 87	*N* = 47	*N* = 40
	*n* (%)	*n* (%)	*n* (%)
Financial incentives			
(Top-up allowances, transport and housing allowance, loans)			
Strongly satisfied	3 (3.45)	3 (6.4)	0
Somewhat satisfied	20 (23.0)	13 (27.7)	7 (17.5)
Satisfied	26 (29.9)	11 (23.40	15 (37.5)
Somewhat dissatisfied	17 (19.5)	11(23.4)	6 (15.0)
Totally dissatisfied	21 (24.1)	9 (19.2)	12 (30.0)
Non-financial retention incentives			
(Residential stands, free accommodation and transport, airtime, lunch and tea)			
Strongly satisfied	1 (12)	1 (2.1)	0
Somewhat satisfied	4 (4.6)	3 (6.4)	1 (2.5)
Satisfied	23 (26.4)	13 (27.7)	10 (25.0)
Somewhat dissatisfied	28 (32.2)	16 (34.0)	12 (30.0)
Totally dissatisfied	31 (35.6)	14 (29.8)	17 (42.5)
Support for post-basic and post-graduate training			
(Support for post-basic and post-graduate training)			
Strongly satisfied	1 (12)	1 (2.1)	0
Somewhat satisfied	6 (6.9)	5 (10.6)	1 (2.5)
Satisfied	17 (19.5)	11(23.4)	6 (15.0)
Somewhat dissatisfied	27 (31.0)	12 (25.5)	15 (37.5)
Totally dissatisfied	36 (41.4)	18 (38.3)	18 (45.0)
On the job training and development			
(On-job training and development)			
Strongly satisfied	15 (17.2)	14 (29.8)	1 (2.5)
Somewhat satisfied	13 (14.9)	9 (19.2)	4 (10.0)
Satisfied	17 (19.50	13 (27.7)	4 (10.0)
Somewhat dissatisfied	24 (27.6)	7 (14.9)	17 (42.5)
Totally dissatisfied	18 (20.7)	4 (8.5)	14 (35.0)
Health welfare			
(Medical aid)			
Strongly satisfied	4 (4.6)	2 (4.3)	0
Somewhat satisfied	24 (27.6)	12 (25.5)	2 (5.0)
Satisfied	34 (39.10	17 (36.2)	12 (30.0)
Somewhat dissatisfied	21 (24.1)	12 (25.5)	17 (42.5)
Totally dissatisfied	4 (4.6)	4 (8.5)	9 (22.5)
Safety welfare			
(Protective clothing and Protocols)			
Strongly satisfied	5 (5.8)	3 (6.4)	2 (5.0)
Somewhat satisfied	29 (33.3)	15 (31.9)	14 (35.0)
Satisfied	33 (37.9)	20 (42.6)	13 (32.5)
Somewhat dissatisfied	17 (19.5)	7 (14.9)	10 (25.0)
Totally dissatisfied	3 (3.5)	2 (4.3)	1 (2.5)
Deployment of adequate staff and workload			
Strongly satisfied	11 (12.60	5 (10.6)	6 (15.0)
Somewhat satisfied	20 (23.0)	10 (21.3)	10 (25.0)
Satisfied	31 (35.6)	17 (36.2)	14 (35.0)
Somewhat dissatisfied	20 (23.0)	13 (27.7)	7 (17.5)
Totally dissatisfied	5 (5.8)	2 (4.3)	3 (7.5)
Equipment and tool of trade			
(Medical equipment and sundries)			
Strongly satisfied	6 (6.90	1 (2.1)	0
Somewhat satisfied	21 (24.1)	12 (25.5)	5 (12.5)
Satisfied	43 (49.4)	26 (55.3)	9 (22.5)
Somewhat dissatisfied	16 (18.4)	7 (14.9)	17 (42.5)
Totally dissatisfied	1 (1.2)	9 (22.5)	9 (22.5)
Salaries			
Strongly satisfied	2 (2.3)	0	0
Somewhat satisfied	1 (1.2)	2 (4.3)	1 (2.5)
Satisfied	22 (25.3)	13 (27.7)	9 (22.5)
Somewhat dissatisfied	28 (32.2)	12 (25.5)	16 (40.0)
Totally dissatisfied	34 (39.1)	20 (42.6)	14 (35.0)

**Fig. 3:**
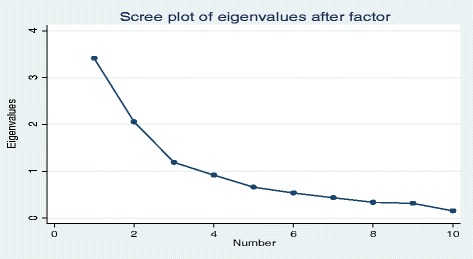
Scree plot

For this, a Scree Plot outlined in Fig. 1 was generated and used to determine the optional number of components/eigenvalues (homogeneous sets/factors) into which variables were clustered (Fig. 3).

The Scree Plot above shows that the last big drop occurs between the third and fourth components, so using the first three components was an easy choice. These three eigenvalues are outlined in Table 5.

**Table 5:** Outline of eigenvalues

Factor analysis/correlation				Number of observations = 87
Method: principal component factors				Retained factors = 3
Rotation: (unrotated)				Number of parameters = 27
Factor	Eigenvalue	Difference	Proportion	Cumulative
Factor 1	3.39	1.32	0.34	0.34
Factor 2	2.07	0.97	0.21	0.55
Factor 3	1.10	0.35	0.11	0.66
Factor 4	0.75	0.05	0.07	0.73
Factor 5	0.70	0.15	0.07	0.80
Factor 6	0.54	0.06	0.05	0.85
Factor 7	0.48	0.07	0.05	0.90
Factor 8	0.41	0.09	0.04	0.94
Factor 9	0.31	0.07	0.03	0.97
Factor 10	0.25		0.03	1.00

LR test: independent vs saturated: *χ* ^2^(45) = 280.17, Prob + *χ* ^2^ = 0.00

Table 6 shows the extracted components of these three eigenvalues/factors.

**Table 6:** The three factors

Factor analysis/correlation				Number of observations = 7
Method: principal component factors				Retained factors = 3
Rotation: orthogonal varimax (Kaiser off)				Number of parameters = 27
Factor	Variance	Difference	Proportion	Cumulative
Factor 1	2.68	0.08	0.27	0.27
Factor 2	2.59	1.30	0.26	0.53
Factor 3	1.29		0.13	0.66

LR test: independent vs. saturated: *χ* ^2^(45) = 280.17, Prob > *χ* ^2^ = 0.0000

They explain nearly 65.6% of the variability in the original ten variables, so one can considerably reduce the complexity of the data set by using these three components, with only 26% loss of information. Nevertheless, the resultant factor loadings are outlined in Table 7.

**Table 7:** Factor loadings

Variable	Factor 1	Factor 2	Factor 3
Financial incentives		0.79	
Non-financial retention incentives	0.48	0.75	
Support for post-basic and post-graduate training		0.66	0.47
On the job training and development	− 0.37	0.77	
Health welfare	0.65		0.47
Safety welfare	0.72		
Deployment of adequate staff and workload	0.77		− 0.32
Deployment of Community Health Volunteers			0.83
Equipment and tool of trade	0.71		
Salaries	0.55	0.50	

Table 7 shows the rotated factor loadings for each function, which is the amount of relationship or amount of contribution by the variable to the new factor. Loadings less than 0.30 are not shown. To help interpret the factor structure, a rotated component matrix was used to help determine what the components represented. From the factor loadings in Table 5, the first rotated factor is most highly positively correlated with the questions on health welfare, safety welfare, deployment of adequate staff and workload, equipment of tool of trade, and salaries. The second factor is most highly correlated with questions on financial incentives, non-financial retention incentives, support for post-basic and post-graduate training, and on-the-job training and development. The third factor is most highly correlated with the question on the deployment of community health workers. Table 8 identifies the three main factors and also provides a summative overview of the most highly correlated interventions.

**Table 8:** Factor names and summative overview of correlation

Factor name	Question	Satisfaction assessment domain	Correlation
Healthcare worker welfare, deployment and equipment	Q17e	Health welfare Safety welfare	0.65
	Q17f	Deployment of adequate staff and workload	0.72
	Q17g	Equipment and tool of trade	0.77
	Q17i		0.71
Incentives and post-basic training	Q17a	Financial incentives	0.79
	Q17b	Non-financial retention incentives	0.75
	Q17c	Support for post-basic and post-graduate training	0.66
	Q17d	On the job training and development	0.77
Deployment of volunteers	Q17h	Deployment of Community Health Volunteers	0.83

The first main factor that healthcare workers were most satisfied with was the deployment of community health workers (CHWs) that had the highest correlation of 0.83. We established that CHWs were recruited and deployed into two main sub-groups namely peer educators and community health volunteers/village health workers. The deployment of CHWs helped mitigate the shortage healthcare worker shortages at the three public clinics and inside the community as revealed by one sister in charge:

The deployment of Peer Educators has helped lessen the workload on Nurses at our clinic. They are helping us in filing, cleaning and other non-medical roles which are facilitating service delivery at this clinic. The Village Health Workers help perform community outreach interventions that include health education, report the health situation in households, and patient follow-ups. It would not have been possible for us to perform these interventions alone because we are shortstaffed.

However, we established that peer educators often experienced stigma from some community members. Peer educators revealed that this stigma emanated from their HIV status and resulted in name calling by some locals who attended clinics where they were deployed. Despite this, we established from community members that the levels of stigma against HIV-positive individuals and their families had significantly reduced as a result of education and knowledge sharing interventions implemented by the local board and the Ministry of Health in partnership with a health non-government organization that operated in this peri-urban area. It was also established that stigma was compounded by the exodus of community volunteers to other jobs and attrition which created shortages. This was further compounded by the lack of equipment and uniforms and unfulfilled promises that included non-payment of an allowance which also undermined motivation [8–10].

Policy result areas in the second main factor included the provision of financial incentives that had a correlation of 0.79, on-the-job training and development which had a correlation of 0.77, followed by deployment of adequate staff with a correlation of 0.77, and the provision of non-financial incentives with a correlation of 0.75. These are policy result areas that yielded the second highest levels of satisfaction amongst healthcare workers in Epworth peri-urban area. We established from healthcare workers at the two municipal clinics and mission clinic that salary top-up allowances, the deployment of more healthcare workers that resulted from the opening of the second municipal clinic in September 2011 and intervention by the Ministry of Health at the other two clinics, and the provision ofresidential stands and accommodation helped revive healthcare worker reform in this peri-urban community [8, 10]. However, we also established that effectiveness of the interventions on human resources for health were undermined by capacity constraints that resulted in failure to pay the salary top-up allowance, provide residential stands and accommodation to all healthcare workers, and deployment of an adequate number of health personnel to meet requirements. The financial constraints that resulted in the inability to pay salary top-up allowances to all health personnel undermined worker motivation as it not only resulted in the failure to address the challenge of inadequate salaries but also left some healthcare workers feeling left out as revealed by one nurse as follows:

Those of us that started working here in 2013 are not receiving those top up allowances. It is demoralising and I cannot get it out of my mind because we do the same job but are treated differently on that. This is compounded by that someone on study leave receive the top up allowance whilst you on duty everyday receive nothing. This is not fair. It hurts and makes you feel unwanted, unappreciated and less special. This is further compounded by that a Nurse Aid who is in a lower grade compared to mine (Registered General Nurse) receives the top up allowance whilst l do not. How does it feel when they call some to go and receive those top up allowances from the same consultation room. We have engaged them on the matter but they seem reluctant to respond and I am not happy about this at all [8–10].

The third factor consisted policy result areas that had the lowest satisfaction amongst healthcare workers in Epworth. These included safety with a correlation of 0.72, equipment and tools of trade with 0.72, health welfare with 0.65, and salaries which had the lowest correlation of 0.55. It was revealed that there were some safety concerns particularly amongst nurse aids who appeared to have limited knowledge about how to use the safety protocol in the event of an accident or emergency in the workplace. We established that this might have emanated from the exclusion of Nurse Aids from regular training workshops, often attended by nurses, where these safety protocols were taught. This was compounded by the lack of adequate financial resources to provide all the required equipment and a subsidized medical aid fund to help healthcare workers in the event that they contract communicable diseases whilst on duty. The unavailability of a subsidized medical aid scheme compounded the situation as healthcare workers were reluctant to join medical aid schemes because of low salaries. Some respondents proposed that there should be a free medical aid scheme or special subsidized medical aid scheme for healthcare workers.

My proposal is that the government should provide either a free or subsidised medical aid scheme to health workers and their families. There should also be a special medical aid arrangement for those working in the TB Department to cover treatment costs in the event that one contracts MDRTB. MDRTB takes up to two years to treat, which is longer than the 90 days of sick leave that we are given. It therefore means that if one contracts it, there is a risk of them either losing their job or be forced risk infecting others by coming to work sick.

Our findings revealed that this was compounded by salaries which had the lowest correlation of 0.55. Despite salaries having been denominated in US dollars (US$) and there no being delays in their payment, healthcare workers revealed that these salaries were not adequate to meet all basic expenses amongst which include transport, food, rentals, clothing, and school fees. This challenge was compounded by lower salaries for healthcare workers in the local private sector and the lack of satisfaction with the salary grading system and failure to pay salary top-up allowances to all healthcare workers at local public clinics.

## Discussion

The engagement of locals towards the recruitment and deployment of community health workers (CHWs) to help complement health workers at clinics in the pursuit of health system in peri-urban areas is our first theme. Our findings revealed that healthcare workers at clinics in Epworth peri-urban community were most satisfied with the deployment of community health workers, which was the most highly correlated outcome with 0.83. The recruitment and deployment of CHWs impacts positively on healthcare workers in peri-urban areas. Not only do CHWs help mitigate healthcare worker shortages at clinics but they are also an important link between the community and health facilities, facilitating the implementation of health interventions in peri-urban areas. A systematic review of the role and outcomes of community health volunteers in HIV care in sub-Saharan Africa revealed that CHWs help to facilitate the implementation of health interventions in the community. For example, in South Africa and Kenya, CHWs helped educate families, primary caregivers, and communities on symptoms and treatment of opportunistic infections, infection control, drug administration and reaction. In South Africa, Zambia, and Mozambique, CHWs help train HIV-positive individuals on antiretroviral treatment (ART) readiness, advantages, and side effects. Additionally, the presence of CHWs was reported to contribute to a positive perception of people living with HIV in the community by demystifying HIV, through interaction with people who had the disease and increasing their social visibility and acceptance [15]. In the hard to reach areas of Myanmar, CHWs supported the work of midwives at local health centers. They also helped in community mobilization for interventions that include immunization, advocating for safe water and sanitation and health education and awareness [16]. The presence of CHWs not only facilitates the implementation of health interventions but also contributes towards improving the equitable distribution, availability, and accessibility of healthcare workers as prescribed by the 2030 Global Health Workforce Strategy in the pursuit of goals 3 and 11 of the 2030 Sustainable Development Agenda and the health sector reform goal of universal health coverage as prescribed through Resolution WHA 67.24 by the World Health Organization [3–5].

However, CHWs in peri-urban areas experience challenges that undermine their effectiveness. For example, peer educators in Epworth complained that they experienced stigma from some locals as a result of their HIV status. This undermines the love and intrinsic desire and passion, often expressed as a calling, for volunteering and public service in local communities. Perhaps important lessons to help avert this can be drawn from the Morogoro Region of Tanzania where moral support through recognition, positive comments, and encouragement by families and communities helped motivate CHWs [17]. This view is supported by Mwai et al. who suggests that recognition and integration into the wider health system help sustain CHWs in sub-Saharan Africa [15].

In Epworth, however, our findings also revealed other compounding factors negatively affecting CHWs. These include the exodus of volunteers to other jobs, attrition, lack of equipment and uniforms, and non-payment of allowances [8, 10]. In Morogoro, Tanzania, whilst the lack of a salary was the main reason why some people either decided not to become CHWs or resigned from it, CHWs cited the receipt of stipends to attend trainings as a motivator that eased the burden of volunteer work and also helped generate support from family members. Some CHWs hoped for future financial gain or employment within the health system, which motivated them to continue volunteering. For other CHWs, however, their hope derived from the receipt of non-monetary material incentives that included training, tools, and supplies to do their work, for example, bicycles, weighing scales, register books, and job aids. This was also helped by some financial and material support from community members in the form of food, help with farm work, and payment for services received [17]. These interventions can also help community health voluntary work in the pursuit of the 2030 Global Health Workforce Strategy, the 2030 Sustainable Development Goals, and universal health coverage in peri-urban areas [3–5].

Human resource for health reform intervention through the provision of financial incentives, basic and post-basic training, deployment of more health personnel, and non-financial incentives to healthcare workers in peri-urban areas is our second theme. Our findings revealed that these interventions were a second main factor that impacted positively on healthcare workers in Epworth peri-urban area. In this category, financial incentives had a correlation of 0.79, on-the-job training and development which had a correlation of 0.77, followed by deployment of more staff with a correlation of 0.77, and the provision of non-financial incentives with a correlation of 0.75. The payment of salary top-up allowances is the main financial incentive often used in healthcare worker reform interventions. In Malawi, salary top-up allowances that were paid to healthcare workers facilitated the implementation of the 6-year Emergency Human Resources Programme that helped alleviate the healthcare worker crisis in 2005 [4]. An almost similar strategy was implemented in Kenya where the Government introduced additional “extraneous” and “non-practicing” allowances for healthcare workers deployed in under-supplied areas. For doctors who entered the public service with a basic wage of KES 11 690 (about US$ 145) per month, the additional allowances in this job group amounted to KES 25 000 (about US$ 311) per month meaning that the wages de facto tripled. According to a key informant in the Ministry of Health of Kenya, this measure attracted 500 doctors that sought employment in the Kenyan public service for deployment in underserved areas [18]. In Epworth, our findings also revealed that salary top-up allowances helped supplement salaries that healthcare workers viewed as inadequate. In turn, this not only contributed towards the retention of healthcare workers but was also a source of motivation which has positive outlook towards the 2030 Global Health Workforce Strategy [5]. However, it must also be noted that the failure to provide financial incentives to all healthcare workers undermine the effectiveness of this reform policy strategy. In Epworth peri-urban area, the failure to pay the salary top-up allowances to all healthcare workers created a sense of division and exclusion and also undermined morale amongst health personnel.

The provision of basic and post-basic training, deployment of more human resources for health personnel, and non-financial incentives may also be used to address human resource health challenges in peri-urban areas. This is in line with findings in rural Mali where it was established that continuous training for rural practice amongst community doctors by the Ministry of Health helped increase self-confidence and self-esteem, overcome the challenge of professional isolation through the provision of a sense of belonging to a professional group sharing a common professional identity, and also reduce the cultural gap. Additionally, follow-up visits and continued training and mentoring also contributed towards their retention [19]. This is compatible with findings from studies in Sierra Leone in which it was revealed that opportunities for on-the-job training and continued professional development help contribute towards healthcare worker motivation and retention [20]. However, it was also established in both studies that training alone are insufficient towards the motivation and retention of retain health personnel. In this regard, it was also proposed that the provision of suitable accommodation, transport, and communication and adequate staffing corresponds positively on workload, and social amenities such as electricity, satellite television, and internet also help address health personnel challenges in resource-constrained communities [19, 20].

Our third theme focused on policy result areas that had impacted less satisfactorily amongst healthcare workers in Epworth. These included safety that had a correlation of 0.72, equipment and tools of trade with 0.72, and health welfare with 0.65. Safety is important in helping protect healthcare workers from accidental disease infection whilst on duty. However, our findings in Epworth suggest that information and knowledge about safety protocols must be disseminated through training to all healthcare workers so as to assure safety for all workers. In addition, it also appears that human resource for health reform interventions in peri-urban communities must also include the provision of either a free medical aid scheme or special subsidized medical aid scheme for healthcare workers, particularly those who work in high-risk areas of healthcare delivery. In Epworth, it appears that this is a necessity for health personnel who work in the TB Department to help them cover treatment costs in the event that they contract MDRTB. To mitigate these challenges, a study in the Philippines proposed interventions that include injury and illness surveillance and frequent training of occupational health nurses that facilitates understanding between workplace factors and injuries and illnesses. This study further proposed that advocacy for occupational health and safety to management at local and national levels for actions that protect workers may also help [21].

The payment of adequate salaries to healthcare workers in peri-urban areas towards human resources for health reform was our fourth theme. The payment of adequate salaries not only helps healthcare workers meet their basic needs but also contributes towards motivation and retention, a key objective of the 2030 Global Health Workforce Strategy [5]. However, our findings revealed that healthcare workers in Epworth peri-urban area were not satisfied with their salaries, which had the lowest correlation of 0.55. This was the policy result area in which healthcare workers were least satisfied in Epworth. Our findings revealed that despite having been denominated in the US dollars (US$), the salaries were not adequate to meet all basic expenses each month. The payment of lower salaries by the local private sector, lack of satisfaction with the salary grading system, and failure to pay salary top-up allowances to all healthcare workers at local public clinics compounded this challenge [4]. In Mali and Sierra Leone, it was established that adequate salaries not only are a source of health personnel motivation but also helps towards their retention in resource-constrained communities [19, 20]. This will help contribute towards motivation, availability, accessibility, and quality of services by health human resources as prescribed by the 2030 Global Health Workforce Strategy, attainment of goals 3 and 11 of the 2030 Sustainable Development Agenda and universal health coverage [3–7].

## Conclusions

Three main factors were used to explore the impact of human resource for health policy interventions on healthcare workers in Epworth. The first factor was the engagement of locals through the deployment of community health workers to help complement health personnel in human resource for health reform intervention. This was also our first theme in which we concluded that healthcare workers in Epworth were most satisfied as reflected by the highest correlated outcome. In our second main factor and theme, it was concluded that the provision of financial incentives, basic and post-basic training, deployment of more health personnel, and non-financial incentives to healthcare workers in peri-urban areas were important human resources for health reform interventions. However, we noted that the success of these interventions depends on the capacity to provide them to all healthcare workers in peri-urban areas. Our third theme focused on policy result areas that had impacted less satisfactorily on healthcare workers in Epworth. We concluded that these included health and safety welfare, and the provision of equipment and tools of trade. It was however concluded that healthcare workers were least satisfied with the inadequate salaries that were paid. In this regard, our overall conclusion is that whilst the payment of adequate salaries, safety and welfare, provision of equipment, and tools of trade are the most important priorities, these interventions alone are insufficient towards overcoming healthcare worker challenges in peri-urban areas. The pursuit of equitable distribution, availability, accessibility, competency, and motivation of healthcare workers in peri-urban areas also requires the deployment of more healthcare workers and community health workers, provision of financial incentives, and basic and post-basic training. Whilst future studies in peri-urban areas may focus on the most important priority interventions, it is important to note that overcoming health personnel challenges will also require analysis of other personnel for health interventions as they also have implications towards attaining of the 2030 Global Health Workforce Strategy, Sustainable Development Goals, and universal health coverage.

## Abbreviations

AAC: Academic Advisory Committee
ART: Antiretroviral treatment
CHWs: Community health workers
ELB: Epworth Local Board
HRH: Human Resources for Health
HSB: Health Services Board
MoH: Ministry of Health
MRCZ: Medical Research Council of Zimbabwe
NGO: Non-government organization
PEs: Peer educators
PMOME: Provincial Medical Office of Mashonaland East
REC: Research Ethics Committee
SDGs: Sustainable Development Goals
SDMO: Seke District Medical Office
UN: United Nations
UP: University of Pretoria
VHWs: Village health workers
ZRP: Zimbabwe Republic Police
